# A unified first-order hyperbolic model for nonlinear dynamic rupture processes in diffuse fracture zones

**DOI:** 10.1098/rsta.2020.0130

**Published:** 2021-05-03

**Authors:** A.-A. Gabriel, D. Li, S. Chiocchetti, M. Tavelli, I. Peshkov, E. Romenski, M. Dumbser

**Affiliations:** ^1^ Ludwig-Maximilians-Universität München, Theresienstr. 41, 80333 München, Germany; ^2^ Sobolev Institute of Mathematics, 4 Acad. Koptyug Avenue, 630090 Novosibirsk, Russia; ^3^ Laboratory of Applied Mathematics, University of Trento, Via Mesiano, 77, 38123 Trento, Italy

**Keywords:** dynamic earthquake rupture, nonlinear large-strain elasto-plasticity, diffuse interface/phase field approach, complex fault and secondary crack topology, multi-physics coupling within fault zones, thermodynamically compatible systems

## Abstract

Earthquake fault zones are more complex, both geometrically and rheologically, than an idealized infinitely thin plane embedded in linear elastic material. To incorporate nonlinear material behaviour, natural complexities and multi-physics coupling within and outside of fault zones, here we present a first-order hyperbolic and thermodynamically compatible mathematical model for a continuum in a gravitational field which provides a unified description of nonlinear elasto-plasticity, material damage and of viscous Newtonian flows with phase transition between solid and liquid phases. The fault geometry and secondary cracks are described via a scalar function *ξ* ∈ [0, 1] that indicates the local level of material damage. The model also permits the representation of arbitrarily complex geometries via a diffuse interface approach based on the solid volume fraction function *α* ∈ [0, 1]. Neither of the two scalar fields *ξ* and *α* needs to be mesh-aligned, allowing thus faults and cracks with complex topology and the use of adaptive Cartesian meshes (AMR). The model shares common features with phase-field approaches, but substantially extends them. We show a wide range of numerical applications that are relevant for dynamic earthquake rupture in fault zones, including the co-seismic generation of secondary off-fault shear cracks, tensile rock fracture in the Brazilian disc test, as well as a natural convection problem in molten rock-like material.

This article is part of the theme issue ‘Fracture dynamics of solid materials: from particles to the globe’.

## 1. Introduction

Multiple scales, multi-physics interactions and nonlinearities govern earthquake source processes, rendering the understanding of how faults slip a grand challenge of seismology [1,2]. Over the last decades, earthquake rupture dynamics have been commonly modelled as a sudden displacement discontinuity across a simplified (potentially heterogeneous) surface of zero thickness in the framework of elastodynamics [3]. Such earthquake models are commonly forced to distinguish artificially between on-fault frictional failure and the off-fault response of rock. Here, we model natural fault damage zones [4,5] by adopting a diffuse crack representation.

In recent years, the core assumption that faults behave as infinitely thin planes has been challenged [6]. Efforts collapsing the dynamics of earthquakes to single interfaces may miss important physical aspects governing fault-system behaviour such as complex volumetric failure patterns observed in recent well-recorded large and small earthquakes [7,8] as well as in laboratory experiments [9]. However, the mechanics of fault and rupture dynamics in *generalized nonlinear visco-elasto-plastic materials* are challenging to incorporate in earthquake modelling. Earthquakes propagate as frictional shear fracture of brittle solids under compression along pre-existing weak interfaces (fault zones), a problem which is mostly unsolvable analytically. For numerical modelling, dynamic earthquake rupture is often treated as a nonlinear boundary condition^1^ in terms of contact and friction, coupled to seismic wave propagation in linear elastic material. The evolving displacement discontinuity across the fault is defined as the earthquake-induced slip. Typically, the material surrounding the fault is assumed to be linear, isotropic and elastic, with all nonlinear complexity collapsed into the boundary condition definition of fault friction (e.g. [11]), which take the form of empirical laws describing shear traction bounded by the fault strength. In an elastic framework, high-stress concentrations develop at the rupture front. The corresponding inelastic off-fault energy dissipation (*off-fault damage*) and its feedback on rupture propagation [12] can be modelled in the form of (visco-)plasticity of Mohr-Coulomb or Drucker–Prager type [13,14], a continuum damage rheology which may account for high strain rate effects [15–17], or explicit secondary tensile and shear fracturing [18–20].

Numerical modelling of crack propagation has been a long-standing problem not only in seismology but also in computational mechanics. Emerging approaches in modelling fracture and rupture dynamics include phase-field and varifold-based representations of cracks to tackle the major difficulty of the introduction of strong discontinuities in the displacement field in the vicinity of the crack. Current state-of-the-art methods in earthquake rupture dynamics [21] require explicit fracture aligned meshing, thus, generally (with recent exceptions [22]) require fractures to be predefined, and typically only permit small deformations. Using highly efficient software implementations of this approach large-scale earthquake modelling is possible [23–25]. Alternative spatial discretizations which allow representing strong discontinuities at the sub-element level, such as the eXtended finite element method (XFEM) [26], introduce singularities when an interface intersects a cell, but are quite difficult to implement in an efficient manner.

In distinction, *diffuse interface approaches* ‘smear out’ sharp cracks via a smooth but rapid transition between intact and fully damaged material states [27–29]. Within various diffuse interface approaches, the most popular one is the *phase-field approach*, which allows us to model complicated fracture processes, including spontaneous crack initiation, propagation, merging and branching, in general situations and for 3D geometries. Critical ingredients of the phase-field formulation are rooted in fracture mechanics, specifically by incorporating a critical fracture energy (from Griffith’s theory [30]), which is translated into the regularized continuum gradient damage mechanics [31]. Several theoretical methods have been recently proposed for shear fracture (e.g. [32] for mode III) which is dominating earthquake processes. Phase-field models have also been successfully applied for brittle fracture in rock-like materials [33] on small scales (mm’s of slip).

The material failure model discussed in this paper also belongs to the class of diffuse interface models in which the damaged material or a crack is considered as another *phase* of the material and represented by a continuous scalar field *ξ* ∈ [0, 1], called the *damage variable*. As in phase-field approaches, a crack or failure front is represented not as a discontinuity of zero thickness but as a *diffuse interface* across which *ξ* changes continuously from 0 (intact material) to 1 (fully damaged material) resulting in gradual but rapid degradation of material stiffness. Despite this conceptual similarity, the model developed here is very different from the phase-field models. An important feature of the phase-field models is the presence of the non-local regularization term $∼||\nabla \phi |{|}^{2}$ in the free energy, with *ϕ* being the phase field. Without such a regularization term, the numerical treatment of a phase-field model is problematic due to numerical instabilities and mesh dependency of the numerical solution. This indicates the ill-posedness of the underlying governing PDEs, e.g. see [34,35]. By contrast, the model developed here originating from [36,37] does not require non-local regularization terms^2^ and is formulated based on the thermodynamically compatible continuum mixture theory [40,41] which results in a *first-order symmetric hyperbolic* governing PDE system and thus is intrinsically well-posed, at least locally in time. Mathematical regularity of the model is supported by the stability of the hereafter presented numerical results, including a mesh convergence analysis (see §3). More generally, the developed model belongs to the class of Symmetric Hyperbolic and Thermodynamically Compatible (SHTC) equations [42–45]. Apart from the PDE type used (the phase-field models are formulated as second-order Allen-Cahn-type [46,47] or fourth-order Cahn-Hilliard-type [48–50] parabolic PDEs), there is also an important conceptual difference between the developed mixture type approach and the phase-field approaches. In the latter, the phase transformation is entirely controlled by the free energy functional, which usually consists of three terms: $\Psi (\varepsilon ,\phi ,\nabla \phi )=\Psi_{1}(\varepsilon ,\phi )+\Psi_{2}(\phi )+\Psi_{3}(\nabla \phi ),$ where **ε** is the small elastic strain tensor, Ψ_1_ is the elastic energy which comprises a degradation function, Ψ_2_ is the damage potential (usually a double-well potential but also single-well potentials are used [51]), and Ψ_3_ is the regularization term. In our approach, only an energy equivalent to Ψ_1_(**ε**, *ϕ*) is used [37,52], while the phase-transition is described in the context of irreversible thermodynamics and is controlled by a dissipation potential which is usually a highly nonlinear function of state variables^3^ [44,53]. Yet, it is important to emphasize that the irreversible terms controlling the damage are *algebraic* source terms (no space derivatives), which do not affect the differential operator of the model. This greatly simplifies the discretization of the differential terms in the governing PDE, but nevertheless requires an accurate and robust stiff ordinary differential equation solver [52,54] for the source terms. Since the governing PDE system of our theory contains only first-order derivatives in space and time, it is possible to use explicit time-stepping in the numerical integration [52]. In contrast, the second- and fourth-order phase-field PDEs require the use of an implicit time discretization [47], which is more difficult to implement and may not have advantage over explicit methods if the time step is dictated by the physical time scales, such as in strongly time-dependent processes, e.g. fracture dynamics and wave propagation. We note that a hyperbolic reformulation of phase-field models is possible as recently proposed in [55].

Alternatively, variational views on fracture mechanics can describe crack nucleation intrinsically without *a priori* failure criteria [56,57]. Accounting for microscopic surface irregularities or line defects can be achieved by combining a sharp interface approach with advanced tools of differential geometry such as *curvature varifolds* [58]. These ideas can be seen as a natural extension of the pioneering Griffith’s theory [30] with cracks being represented almost everywhere by differentiable surfaces and evolving Griffith’s energies to account for curvature effects. In this context, we remark that the model presented here is by no means a complete fracture model. In specific situations requiring a very accurate prediction of the fracture process the merely constitutive capabilities of the present model may not be sufficient. Instead, accounting explicitly for the energy accumulating at the irregularities of the crack surface (e.g. at corners and cusps) or the dynamics of microscopic defects near the crack tip might be required. In the first-order hyperbolic diffuse interface framework presented here, this can be achieved by taking into account higher gradients of the state variables such as curvature and torsion in the form of independent state variables [38,39].

## 2. Mathematical model

The continuum model for damage of solids employed in this paper consists of two main ingredients. The first ingredient is the damage model proposed by Resnyansky, Romenski and co-authors [36,37] which is a continuous damage model with a chemical kinetics-type mechanism controlling the damage field *ξ* ∈ [0, 1] (*ξ* = 0 corresponds to the intact and *ξ* = 1 to the fully damaged state), which is interpreted as the concentration of the damaged phase. Being a relaxation-type approach, it provides a rather universal framework for modelling brittle and ductile fracture from a unified non-equilibrium thermodynamics viewpoint, according to which these two types of fractures can be described by the same constitutive relations (relaxation functions), but have different characteristic time scales, e.g. [52]. The second ingredient is the Eulerian finite strain elastoplasticity model developed by Godunov and Romenski in the 1970s [59–61]. It was recently realized by Peshkov & Romenski [62] that the same equations can also be applied to modelling viscous fluid flow, as demonstrated by Dumbser *et al.* in [63] and thus, this model represents a unified formulation of continuum fluid and solid mechanics. In the following, we shall refer to it as the Godunov–Peshkov–Romenski (GPR) model. Being essentially an inelasticity theory, the GPR model provides a unified framework for continuous modelling of potentially arbitrary rheological responses of materials, and in particular of inelastic properties of the damaged material. This, in turn, can be used for modelling of complex frictional rheology in fault zones in geomaterials, see §3. For further details on the GPR model, the reader is referred to [45,62–65]. Our diffuse interface formulation for moving nonlinear elasto-plastic solids of arbitrary geometry and at large strain is given by the following PDE system in Eulerian coordinates:

2.1*a* $$\begin{array}{rl} & \partial_{t}\alpha +v_{k}\partial_{k}\alpha =0,\;\partial_{t}\bar{\rho }+\partial_{k}(\bar{\rho }v_{k})=0, \end{array}$$

2.1*b* $$\begin{array}{rl} & \partial_{t}(\bar{\rho }v_{i})+\partial_{k}(\bar{\rho }v_{i}v_{k}+\alpha p\delta_{ik}-\alpha \sigma_{ik})=\bar{\rho }g_{i}, \end{array}$$

2.1*c* $$\begin{array}{rl} & \partial_{t}A_{ik}+\partial_{k}(A_{im}v_{m})+v_{m}(\partial_{m}A_{ik}-\partial_{k}A_{im})=-\theta_{1}^{-1}(\tau_{1})E_{A_{ik}}, \end{array}$$

2.1*d* $$\begin{array}{rl} & \partial_{t}J_{k}+\partial_{k}(v_{m}J_{m}+T)+v_{m}(\partial_{m}J_{k}-\partial_{k}J_{m})=-\theta_{2}^{-1}(\tau_{2})E_{J_{k}}, \end{array}$$

2.1*e* $$\begin{array}{rl} & \partial_{t}\xi +v_{k}\partial_{k}\xi =-\theta E_{\xi }, \end{array}$$

2.1*f* $$\begin{array}{rl} & \partial_{t}(\bar{\rho }S)+\partial_{k}(\bar{\rho }Sv_{k}+\bar{\rho }E_{J_{k}})=\bar{\rho }\;{\left(\alpha T\right)}^{-1}(\theta_{1}^{-1}E_{A_{ik}}E_{A_{ik}}+\theta_{2}^{-1}E_{J_{k}}E_{J_{k}}+\theta E_{\xi }E_{\xi })\geq 0, \end{array}$$

2.1*g* $$\begin{array}{rl} & \partial_{t}(\bar{\rho }E)+\partial_{k}(v_{k}\bar{\rho }E+v_{i}(\alpha p\delta_{ik}-\alpha \sigma_{ik})+q_{k})=\bar{\rho }g_{i}v_{i}, \end{array}$$

where we use the Einstein summation convention over repeated indices and ∂_*t*_ = ∂/∂*t*, ∂_*k*_ = ∂/∂*x*_*k*_. Here, (2.1*a*)_1_ is the evolution equation for the colour function *α* that is needed in the diffuse interface approach (DIM) as introduced in [64,66] for the description of solids of arbitrary geometry (*α* = 1 inside of the solid body and *α* = 0 outside); $\bar{\rho }=\alpha \rho$ and (2.1*a*)_2_ is the mass conservation law with *ρ* being the material density; (2.1*b*) is the momentum conservation law and *v*_*i*_ is the velocity field; (2.1*c*) is the evolution equation for the *distortion field* ***A*** = [*A*_*ik*_], which is the main field in the GPR model and can be viewed as the field of *local basis triads*^4^ representing the deformation and orientation of an infinitesimal material element [39,62,63]; (2.1*d*) is the evolution equation for the specific thermal impulse *J*_*k*_, describing the heat conduction in the matter via a hyperbolic (non Fourier–type) model; (2.1*e*) is the evolution equation for the material damage variable *ξ* ∈ [0, 1], where *ξ* = 0 indicates fully intact material and *ξ* = 1 fully damaged material. Finally, (2.1*f*) is the entropy evolution equation with the positive source product on the right-hand side (second law of thermodynamics) and (2.1*g*) is the energy conservation law (first law of thermodynamics). Other thermodynamic parameters are defined via the total energy potential *E* = *E*(*ρ*, *S*, ***v***, ***A***, ***J***, *ξ*): $p=\rho^{2}E_{\rho }$ is the thermodynamic pressure, $\sigma =[\sigma_{ik}]=[\sigma_{ik}^{e}+\sigma_{ik}^{t}]$ is the stress tensor with contributions to the mechanical stress due to tangential $\left[\sigma_{ik}^{e}=-\rho A_{ji}E_{A_{jk}}\right]$ and thermal stress $\left[\sigma_{ik}^{t}=\rho J_{i}E_{J_{k}}\right]$ (note that **σ**^*e*^ in not necessary trace-free), and *T* = *E*_*S*_ is the temperature. The total mechanical stress tensor is defined as $\Sigma =[\Sigma_{ik}]=[-p\delta_{ik}+\sigma_{ik}^{e}]$, where *δ*_*ik*_ is the Kronecker delta. With a state variable in the subscript of the energy, we denote partial derivatives, e.g. $E_{\rho }=\partial E/\partial \rho$, $E_{A_{ij}}=\partial E/\partial A_{ij}$, etc. The heat flux is defined as *q*_*k*_, and *g*_*i*_ is the gravitational acceleration vector. Also, because we are working in an Eulerian frame of reference, we need to add transport equations of the type ∂_*t*_*λ* + *v*_*k*_∂_*k*_*λ* = 0 to the above evolution equations for all the material parameters (e.g. Lamé constants) in case of heterogeneous material properties, see [52].

In order to close the system one must specify the total energy potential as a function of the state variables, i.e. *E* = *E*(*ρ*, *S*, ***v***, ***A***, ***J***, *ξ*). This potential then generates the fluxes (reversible time evolution) and source terms (irreversible time evolution) by means of its partial derivatives (thermodynamic forces) with respect to the state variables. Here, we make the choice *E* = *E*_1_ + *E*_2_ + *E*_3_, decomposing the energy into a contribution from the microscale *E*_1_, the mesoscale *E*_2_ and the macroscale *E*_3_. The individual contributions read as follows:

2.2 $$E_{1}=\frac{K}{2\rho_{0}}{\left(1-\rho /\rho_{0}\right)}^{2}+c_{v}T_{0}\left(\frac{\rho }{\rho_{0}}\right)(e^{S/c_{v}}-1)+H(T-T_{c})h_{c},$$

where *ρ*_0_ and *T*_0_ are the reference mass density and temperature, *h*_*c*_ is the latent heat, *T*_*c*_ is the critical temperature at which phase transition occurs, *H*(*T*) is the Heaviside step function, *c*_*v*_ is the heat capacity at constant volume. As a proof of concept, we added the last term in (2.2) and present a demonstration example of the model’s capability to deal with solid-fluid phase transition (melting/solidification) in electronic supplementary material, S5. Yet, this corresponds to a simplified (time-independent) modelling of phase transition and will be improved in the future. Also, $K(\xi )=\lambda (\xi )+\frac{2}{3}\mu (\xi )$ is the bulk modulus, *λ*(*ξ*) and *μ*(*ξ*) are the two Lamé constants that are functions of the damage variable *ξ* specified, following [36], as

2.3 $$\lambda (\xi )=\frac{K_{I}K_{D}}{\tilde{K}}-\frac{2\mu_{I}\mu_{D}}{3\tilde{\mu }},\;\mu (\xi )=\frac{\mu_{I}\mu_{D}}{\tilde{\mu }},$$

where the subscripts *I* and *D* denote *intact* and *damaged*, respectively, $K_{I}=\lambda_{I}+\frac{2}{3}\mu_{I}$, $K_{D}=\lambda_{D}+\frac{2}{3}\mu_{D}$, $\tilde{K}=\xi K_{I}+(1-\xi )K_{D}$, $\tilde{\mu }=\xi \mu_{I}+(1-\xi )\mu_{D}$, and it is assumed that the elastic moduli of the intact material *λ*_*I*_, *μ*_*I*_ and of the fully damaged material *λ*_*D*_, *μ*_*D*_ are known.

The macro-scale energy is the specific kinetic energy $E_{3}=\frac{1}{2}v_{i}v_{i}$. Finally, *E*_2_ reads

2.4 $$E_{2}=\frac{1}{4}c_{s}^{2}{\overset{\circ }{G}}_{ij}{\overset{\circ }{G}}_{ij}+\frac{1}{2}c_{h}^{2}J_{i}J_{i},$$

where $c_{s}(\xi )=\sqrt{\mu (\xi )/\rho_{0}}$ is the shear sound speed and *c*_*h*_ is related to the speed of heat waves in the medium (also called the second sound [67], or the speed of phonons). ${\overset{\circ }{G}}_{ik}=G_{ik}-\frac{1}{3}G_{jj}\;\delta_{ik}$ is the deviator of the Finger (or metric) tensor *G*_*ik*_ = *A*_*ji*_ *A*_*jk*_ that characterizes the elastic deformation of the medium.

The dissipation in the system includes three irreversible processes that raise the entropy: the strain relaxation (or shear stress relaxation) characterized by the scalar function *θ*_1_(*τ*_1_) > 0 in (2.1*c*) depending on the relaxation time *τ*_1_, the heat flux relaxation characterized by *θ*_2_(*τ*_2_) > 0 in (2.1*d*), depending on the relaxation time *τ*_2_, and the chemical kinetics like process governing the transition from the intact to damaged state and controlled by the function *θ* in (2.1*e*).

The main idea of the diffuse interface approach to fracture is to consider the material element as a *mixture* of the *intact* and the *fully damaged* phases. These two phases have their own independent material parameters and closure relations, such as functions characterizing the rate of strain relaxation. The strain relaxation approach in the framework of the unified hyperbolic continuum mechanics model [62,63] represented by the evolution equation for the distortion field ***A*** allows us to assign potentially arbitrary rheological properties to the damaged and intact states. In particular, the intact material can be considered as an elastoplastic solid, while the damaged phase can be a fluid, e.g. a Newtonian fluid (see §3c) or viscoplastic fluid, which can be used for modelling of in-fault friction, for example. Yet, in this paper, we do not use an individual distortion evolution equation for each phase, but employ the mixture approach [36,37], and use a single distortion field representing the local deformation of the mixture element, while the individual rheological properties of the phases are taken into account via the dependence of the relaxation time *τ*_1_ on the damage variable *ξ* as follows:

2.5 $$\tau_{1}={\left(\frac{\left(1-\xi \right)}{\tau_{I}}+\frac{\xi }{\tau_{D}}\right)}^{-1},$$

where *τ*_*I*_ and *τ*_*D*_ are shear stress relaxation times for the intact and fully damaged materials, respectively, which are usually highly nonlinear functions of the parameters of state. The particular choice of *τ*_*I*_ and *τ*_*D*_ that is used in this paper reads

2.6 $$\tau_{I}=\tau_{I0}\exp(\alpha_{I}-\beta_{I}(1-\xi )Y),\;\tau_{D}=\tau_{D0}\exp(\alpha_{D}-\beta_{D}\xi Y),$$

where *Y* is the equivalent stress, while *τ*_*I*0_, *α*_*I*_, *β*_*I*_, *τ*_*D*0_, *α*_*D*_, *β*_*D*_ are material constants. In this work, the stress norm *Y* is computed as

2.7 $$Y=A\;Y_{s}+B\;Y_{p}+C,$$

where $Y_{s}=\sqrt{3\;\mathrm{tr}(\mathrm{dev}\Sigma \;\mathrm{dev}\Sigma )/2}$, with dev**Σ** = **Σ** − (tr**Σ**/3)***I***, is the von Mises stress and *Y*_*p*_ = tr**Σ**/3 accounts for the spherical part of the stress tensor. The choice *A* = 1, *B* = *C* = 0, gives *Y* = *Y*_*s*_, that is, the von Mises stress, while other choices of coefficients in equation (2.7) are intended to describe a Drucker–Prager-type yield criterion.

Note that to treat the damaged state as a Newtonian fluid, it is sufficient to take *τ*_*D*_ = const ≪ 1, see §3c or [63]. Non-Newtonian rheologies can also be considered if the proper function for *τ*_*D*_(*Y*) is provided, e.g. see [65]. Thus, the function *θ*_1_ = *τ*_1_ *c*_*s*_(*ξ*)^2^/3 |***A***|^−5/3^ is taken in such a way as to recover the Navier–Stokes stress tensor with the effective shear viscosity $\eta =(1/6)\rho_{0}\tau_{1}c_{s}^{2}$ in the limit *τ*_1_ ≪ 1 [63] and is used for modelling of a natural convection problem in §3c. A pure elastic response of the intact material, as used as fault host rock in §3a cases (i) and (ii), corresponds to *τ*_*I*_ = ∞. By this means, all numerical examples presented throughout §3 follow the rheological formulation given by *θ*_1_ with varying parametrization.

The transition from the intact to the fully damaged state is governed by the damage variable *ξ* ∈ [0, 1] satisfying the kinetic-type equation (2.1*e*), $\dot{\xi }=-\theta E_{\xi }$, with the source term depending on the state parameters of the medium (pressure, stress and temperature). In particular, the rate of damage *θ* is defined as

2.8 $$\theta =\theta_{0}(1-\xi )(\xi +\xi_{\epsilon })\left[(1-\xi ){\left(\frac{Y}{Y_{0}}\right)}^{a}+\xi \left(\frac{Y}{Y_{1}}\right)\right],$$

where $\xi_{\epsilon }$, *Y*_0_ and *Y*_1_, *a* are constants. $\xi_{\epsilon }$ is usually set to $\xi_{\epsilon }=10^{-16}$ in order to trigger the growth of *ξ* with the initial data *ξ* = 0. We note that similar to the chemical kinetics, the constitutive functions of the damage process drive the system towards an equilibrium that is not simply defined as $E_{\xi }=0$, but as $\theta E_{\xi }=0$, e.g. [53]. As a result, the overall response of the material subject to damage (i.e. its stress–strain relation, see also [52]) is defined by the interplay of both irreversible processes; (i) the degradation of the elastic moduli controlled by (2.8) and (ii) the inelastic processes in the intact and damaged phases controlled by (2.5) and (2.6). In the numerical experiments carried out in §3b, the damage kinetics *ξ* also strongly couple with strain relaxation effects, by means of equation (2.5). The function *θ*_2_, governing the rate of the heat flux relaxation, is taken as $\theta_{2}(\tau_{2})=\tau_{2}(c_{h}^{2}/\rho T)$ that yields the classical Fourier law of heat conduction with the thermal conductivity coefficient $\kappa =\tau_{2}c_{h}^{2}$ in the stiff relaxation limit (*τ*_2_ → 0), see [63]. For simplicity, the thermal parameters of the intact and damaged phases are here assumed identical.

Finally, we remark that the problem of parameter selection for our unified model of continuum mechanics is a non-trivial task. Due to the large amounts of parameters, the problem may need to be solved monolithically via numerical optimization algorithms applied to data obtained from observational benchmarks such as triaxial loading experiments. Nonetheless, in certain limiting cases, some rationale can be developed in order to estimate parameters without empirically considering several trial choices. For example, brittle materials can be constructed by choosing a very high value for the exponent *a* in equation (2.6). By this means, the rate of growth *θ* of the damage variable *ξ* will activate as a switch when *Y* reaches the *Y*_0_ threshold. In this specific case, *Y*_0_ can be chosen equivalently to a yield stress. Also, the sensitivity to tensile stresses can be modelled by resorting to techniques that are routinely used in science and engineering, e.g. using the Drucker–Prager yield criterion to compute *Y*. In the Brazilian tensile fracture example in §3b, *β*_*I*,*D*_ are set to zero as the complex stress-dependent mechanisms they control are not necessary for achieving the desired material behaviour. Controlling the relaxation time of the damaged state (*τ*_*D*_) can be useful for modelling friction within a natural fault zone: if a very low relaxation time is chosen, which can be easily achieved by setting *τ*_*D*0_ = 10^−6^s, *α*_*D*_ = *β*_*D*_ = 0, the fault will exert no tangential stresses on the surrounding intact rock, as if it were filled with an inviscid fluid. Specific frictional regimes and (time-dependent) plastic effects can be described by properly choosing the relaxation times *τ*_*I*,*D*_ (via *τ*_*I*0,*D*0_, *α*_*I*,*D*_, *β*_*I*,*D*_), which in general may require more complex automatic optimization strategies.

## 3. Numerical examples

In this section, we present a variety of numerical applications of the GPR model relevant for earthquake rupture and fault zones. The governing PDE system (2.1) is solved using the high performance computing toolkit *ExaHyPE* [68], which employs an arbitrary high-order derivative (ADER) discontinuous Galerkin (DG) finite-element method in combination with an *a posteriori* subcell finite volume limiter on space time adaptive Cartesian meshes (AMR). For details, the reader is referred to [52] and to [63,64,69–73] and references therein.

### (a) Earthquake shear fracture across a diffuse fault zone

**Figure 1.**
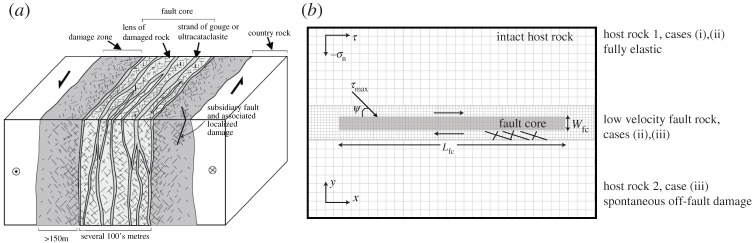
(*a*) Typical strike-slip fault zone structure showing a multiple fault core with associated damage zone in a quartzofeldspathic country rock (from [5]). (*b*) Sketch of the GPR model setup for 2D in-plane right-lateral shear fracture under compression used throughout §3a. In light grey, we depict the prescribed fault core of length *L*_fc_ and width *W*_fc_ which is fully damaged (*ξ* = 1) and embedded in intact host rock (*ξ* = 0). The material properties and rheology of the host rock and fault core differ and are detailed in electronic supplementary material, tables S1 and S2. Grey lines illustrate the initial mesh refinement, which can dynamically adapt as detailed in electronic supplementary material, table S3.

In the following, we explore the GPR diffuse fault zone approach extending the modelling of dynamic earthquake rupture beyond treatment as a discontinuity in the framework of elastodynamics. Figure 1 illustrates the model set-up corresponding to the geological structure of a typical strike-slip fault zone. Dynamic rupture within the ‘fault core’ is governed by a friction-like behaviour achieved by time-dependent modulation of the shear relaxation time *τ*_*D*_ of the fault core’s fully damaged material. At the onset of frictional yielding, the shear relaxation time (*τ*_*D*_) decreases exponentially as in (2.6) with a time-dependent $\beta_{D}^{\prime }$. The temporal evolution of $\beta_{D}^{\prime }$ is modulated at a constant rate during rupture as $\beta_{D}^{\prime }(t)=\beta_{D}\min\left(1,\max\left(0,1-C_{1}\;t\right)\right)$ where *C*_1_ and *β*_*D*_ are constant. Visco-elastic slip accumulates across the diffuse fault core coupled to either fully elastic wave propagation or Drucker–Prager type damage in the host rock.

**(i) Kinematic self-similar Kostrov-like crack.** We first model a kinematically driven *non-singular self-similar shear crack* analog to Kostrov’s solution for a singular crack [74] to study the relation between fault slip, slip rate and shear stress in comparison to traditional approaches, while imposing tractions here avoids the full complexity of frictional rupture dynamics. The 2D set-up (e.g. [75]) assumes a homogeneous isotropic elastic medium (electronic supplementary material, table S2, $c_{s}=c_{p}/\sqrt{3}$), and a pre-assigned fault interface loaded by initial normal stress *σ*_*n*_ = 40 MPa and shear stress *τ* = 20 MPa. An in-plane right-lateral shear crack is driven by prescribing the (sliding) friction *μ*_*f*_ as linearly time-dependent weakening: *μ*_*f*_(*x*, *t*) = max{*f*_*d*_, *f*_*s*_ − (*f*_*s*_ − *f*_*d*_)(*v*_*r*_ *t* − |*x*|)/*R*_*c*_}, with process zone size *R*_*c*_ = 250 m, rupture speed *v*_*r*_ = 2000 m s^−1^, static friction *f*_*s*_ = 0.5 and dynamic friction *f*_*d*_ = 0.25. We empirically find that choosing *C*_1_ = 10 reproduces the propagating shear crack in the reference solution. Thus, $\beta_{D}^{\prime }$ evolves linearly from *β*_*D*_ to 0 during rupture.

**Figure 2.**
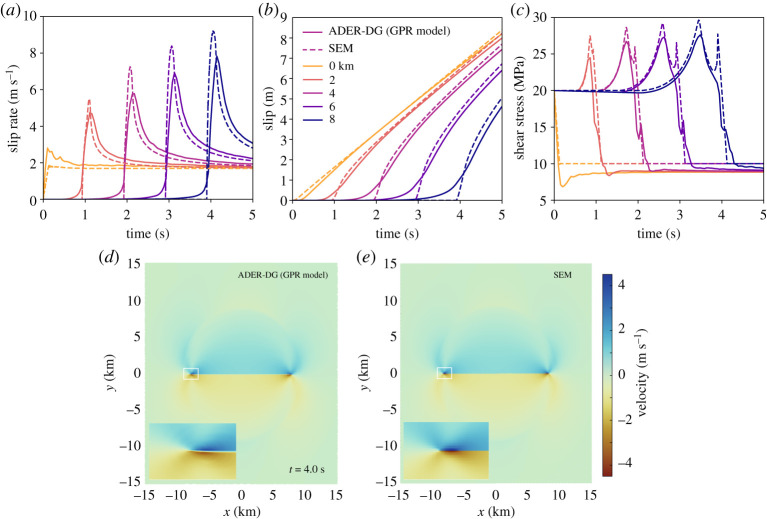
Comparison of the self-similar Kostrov-like crack of the diffuse GPR model (ADER-DG, *p* = 6, *W*_fc_ = 100 m, *L*_fc_ = 20 km, fault core and host rock material are ‘host rock 1’, static AMR) with the discrete fault spectral element *SEM2DPACK* (*p* = 6, *h* = 100 m) solution; (*a*) slip rate, (*b*) slip and (*c*) shear stress time series at increasing hypocentral distances, (*d*,*e*) velocity wavefield at *t* = 4 s (see also Animation S1), and zoom into the rupture tip. (Online version in colour.)

We assume a fully damaged fault core (*ξ* = 1) of prescribed length *L*_fc_ = 20 km and width *W*_fc_ = 100 m embedded in a continuum material resembling intact elastic rock (*ξ* = 0) as illustrated in figure 2*a*. Both, the fault core and the surrounding host rock are treated as the same continuum material besides their differences in *ξ*. The GPR specific material parameters are detailed as ‘host rock 1’ (here, *λ*_*D*_ = *λ*_*I*_, *μ*_*D*_ = *μ*_*I*_) in electronic supplementary material, table S1. The model domain is of size 70 × 70 km bounded by Dirichlet boundary conditions and employs a statically refined mesh surrounding the fault core. The domain is discretized into hierarchical Cartesian computational grids, spaced *h* = 2800 m at the coarsest level, and *h* = 311 m at the second refinement level (electronic supplementary material, table S3). We use polynomial degree *p* = 6 and the subcell finite volume limiter counts 2 *p* + 1 = 13 subcells in each spatial dimension. Figure 2*a*–*c* compares slip, slip rate and shear traction during diffuse crack propagation modelled with the GPR model to a spectral element solution assuming a discrete fault interface spatially discretized with *h* = 100 m with *SEM2DPACK* [76]. The GPR model analog captures the kinematics (i.e. stress drop and fault slip) of the self-similar singular Kostrov crack as well as the emanated seismic waves (figure 2*d*,*e* and Animation S1), while introducing dynamic differences on the scale of the diffuse fault (zoom-in in figure 2*d*). Slip velocity (figure 2*a*) remains limited in peak, similar to planar fault modelling with off-fault plastic deformation [77]. Fault slip (figure 2*b*) appears smeared out at its onset, yet asymptotically approaches the classical Kostrov crack solution. Similarly, shear stresses (figure 2*c*) appear limited in peak and more diffuse, specifically with respect to the secondary peak associated with the passing rupture front. Importantly, (dynamic) stress drops are comparable to the expectation from fracture mechanics for a plane shear crack (even though peak and dynamic level appear shifted). At the crack tip, we observe an initial out-of-plane rotation within the fault core leading to a localized mismatch in the hypocentral region and at the onset of slip across the fault. The GPR model approaches the analytical solution, as illustrated for increasing polynomial degree *p* in electronic supplementary material, figure S1.

**Figure 3.**
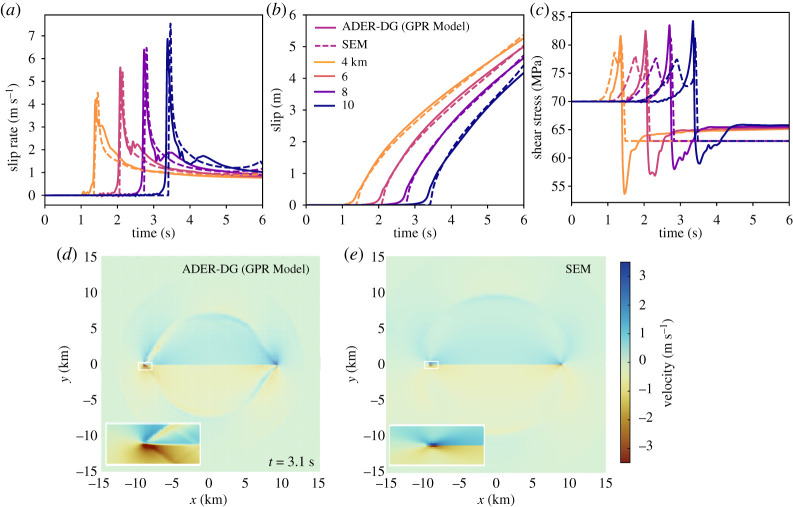
Computational results for the 2D TPV3 dynamic rupture problem. Comparison of the diffuse interface GPR model (ADER-DG, *p* = 6, *W*_fc_ = 100 m, *L*_fc_ = 30 km, fault core of ‘low velocity fault rock’ embedded in ‘host rock 1’, static AMR) with the discrete fault spectral element *SEM2DPACK* solution (*p* = 6, *h* = 100 m), with (*a*) slip rate, (*b*) slip and (*c*) shear stress time series at increasing hypocentral distance. (*d*,*e*) Radiated seismic wavefield in terms of particle velocity at *t* = 3.1 s (see also Animation S2). Zoom-in the crack tips highlights dynamic rupture complexity within the low-rigidity fault core. (Online version in colour.)

**(ii) Spontaneous dynamic rupture.** We next model spontaneous dynamic earthquake rupture in a 2D version [75] of the benchmark problem TPV3 [21] for elastic spontaneous rupture propagation defined by the Southern California Earthquake Center. Our setup resembles the kinematic model (figure 1*a*) including the time-dependent choice of $\beta_{D}^{\prime }(t)$ with *C*_1_ = 10 with an important distinction: we assume a *low-rigidity fault core* (‘low velocity fault rock’ in electronic supplementary material, table S1) by setting P-wave and S-wave velocity in the fault core 30% lower, i.e. *λ*(*ξ*) and *μ*(*ξ*) are decreased by 50%, with respect to the intact rock. A 30% reduction of seismic wave speeds matches natural fault zone observations. The thickness of the low velocity fault rock unit equals the thickness of the fault core itself where *ξ* = 1. The surrounding country rock is again parameterized as fully elastic with the ‘host rock 1’ GPR parametrization (electronic supplementary material, table S1). The fault core is *L*_fc_ = 30 km long and *W*_fc_ = 100 m wide, the domain size is 40 × 40 km, initial loading is *σ*_*yy*_ = −120 MPa and *σ*_*xy*_ = 70 MPa. The computational grid is spaced *h* = 1600 m at the coarsest level, and *h* = 177 m at the second refinement level (electronic supplementary material, table S3). Figure 3 compares, similar to the kinematic case, the diffuse low-rigidity fault ADER-DG GPR results to an elastic discrete fault interface spectral element solution. Fault slip rates (figure 3*a*) are limited in peak and are now clearly affected by smaller scale dynamic complexity, e.g. out-of-plane crack rotation and wave reflections, within the fault core. Fault slip (figure 3*b*) asymptotically resembles the discontinuous, elastic solution. Shear stresses (figure 3*c*) are smeared out and shifted, but capture (dynamic) stress drops, similar to the kinematic model in (i). We note that residual shear stress levels remain higher potentially reflecting oblique shear developing within the diffuse fault core and/or viscous behaviour within the fault core. The diffuse fault core slows down the emitted seismic waves, while amplifying sharp velocity pulses (figure 3*d*,*e* and Animation S2) aligning with observational findings [78]. The GPR model successfully resembles frictional *linear-slip weakening* behaviour [79] within the fault core by defining: *μ*_*f*_(*x*, *t*) = max{*f*_*d*_, *f*_*s*_ − (*f*_*s*_ − *f*_*d*_)*δ*(*x*, *t*)/*D*_*c*_}, with slip-weakening distance *D*_*c*_ = 0.4 m, *f*_*s*_ = 0.677 and *f*_*d*_ = 0.525 similar to the discrete fault solution, *δ*(*x*, *t*) denotes here the diffuse slip within the fault core and is measured as the difference of displacements at its adjacent boundaries. Rupture is not initiated by an overstressed patch, which would be inconsistent with deforming material, but as a kinematically driven Kostrov-like shear-crack with *v*_*r*_ = 4000 m s^−1^ and within a nucleation time of *t* = 0.5 s. In the diffuse model, introducing the low velocity fault rock material within the fault core is required to limit rupture speed while resembling slip rate, slip and stress evolution of the discrete reference model. We conclude that the *rheological fault core properties*, and not the friction law, control important crack dynamics such as rupture speed in our diffuse interface modelling, cf. [80]. A comparison of results assuming a further reduction of fault rock wave speeds to 37% is discussed in the electronic supplementary material.

**Figure 4.**
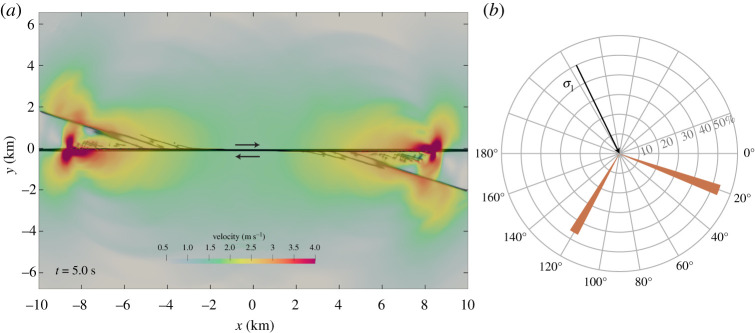
Off-fault shear cracks spontaneously generated in the extensional quadrants of dynamic earthquake rupture (TPV3) in the GPR model (ADER-DG,*p* = 6, *W*_fc_ = 100 m, *L*_fc_ = 20 km, fault core of ‘low velocity fault rock’ embedded in Drucker-Prager type ’host rock 2’, dynamic AMR). (*a*) Velocity wavefield at *t* = 5.0 s (see also Animation S3). Dark colours represent the damage variable *ξ* illustrating the fault core initialized as fully damaged (cf. figure 2*a*) and the propagating secondary off-fault cracks. (*b*) Polar diagram of the statistical orientation of off-fault shear cracks. The two dominant orientations are ≈20^°^ and ≈120^°^. The maximum compressive stress (*σ*_1_) has an orientation angle of 65.3^°^. (Online version in colour.)

**(iii) Dynamically generated off-fault shear cracks.** Localized shear banding is observed in the vicinity of natural faults spanning a wide spectrum of length scales [5], and contributes to the energy balance of earthquakes. We model dynamically generated off-fault shear cracks by combining the spontaneous dynamic rupture model embedded in ‘low velocity fault rock’ with ‘host rock 2’ outside the fault core (electronic supplementary material, table S1, *μ*_*D*_ = 0.8571 *μ*_*I*_, *λ*_*D*_ = *λ*_*I*_ + 0.6667 (*μ*_*I*_ − *μ*_*D*_) in (2.3)). ‘Host rock 2’ is governed by Drucker–Prager yielding [14,81,82] as given by equation (2.7), with $A=1/\sqrt{3}$, *B* = sin (*π*/18), and *C* = −cos (*π*/18) · 95 MPa. The model domain size is 20 × 15 km spatially discretized with *h* = 800 m at the coarsest mesh level (electronic supplementary material, table S3). We here use *dynamic adaptive mesh refinement* (AMR) with two refinement levels and refinement factor r=3 to adapt resolution in regions where the material is close to yielding. The finest spatial discretization is *h* = 89 m. Figure 4*a* illustrates spontaneous shear-cracking in the *extensional* quadrants of the main fault core, where the passing rupture induces a dynamic bimaterial effect [83]. While previous models [14] based on ideal plasticity without damage accumulation numerically capture the formation of single sets of shear bands in Drucker–Prager type off-fault material induced by dynamic rupture propagation across a main fault, we here observe the formation of two conjugate sets of shear fractures: cracks are distributed around two favourable orientations (figure 4*b*). Spacing and length of these shear deformation bands [19,84] may depend on GPR material parametrization (*Y*_0_, *β*_*D*_, cohesion, internal friction angle, etc. see electronic supplementary material, table S1 and [52]) as well as on the computational mesh and will motivate future analysis, as in §3b. High particle velocity is associated with the strong growth of off-fault shear stresses near the fault tip shifting from the propagation direction of the main rupture [85]. We observe the dynamic development of interlaced conjugate shear faulting (Animation S3) resembling recent high-resolution imaging of earthquakes [8].

### (b) Crack formation in a rock-like disc

**Figure 5.**
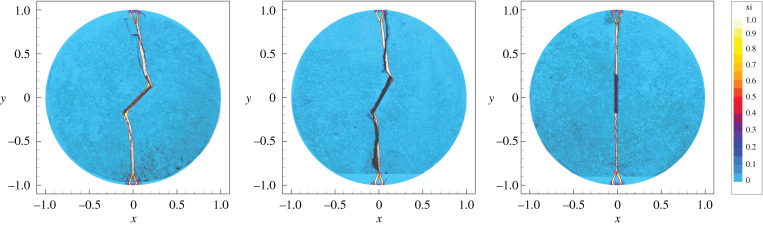
Crack formation in a rock-like disc under vertical load (Brazilian test) for different angles of the pre-damaged area. Comparison of the contour colours of the damage variable *ξ* obtained in the numerical simulations of the GPR model with the cracks observed in experiments. The simulation results are overlaid on top of the photographs from [86]. From left to right: 45^°^, 60^°^ and 90^°^. Only the regions of the disc where *α* > 0.5 are shown. (Online version in colour.)

The GPR framework can be applied to capture tensile fracture, important for earthquake nucleation processes and the microscale of fault zone fracture and damage. We now show that our model is able to correctly describe the fracture mechanisms observed in laboratory settings. Specifically, we reproduce the experimental results of [86] which involve the compression of a rock disc along its diameter (a so-called Brazilian test). In this case, the disc presents a central slit with a given orientation, which influences the early stages of the failure of the rock sample. The test is carried out in two space dimensions on a square computational domain centred at the origin and with side length 2.2 units. The interface of the disc is defined by setting *α* = 0 outside of the unit-radius circle, without requiring a boundary-fitted mesh. The material used in this test has been derived as a weakened variant of a granite-like brittle rock. In particular, it replicates the strong difference in shear resistance found under compression or tensile loads. The material is characterized by the following choice of parameters: *ρ* = 2620 kg m^−3^, *μ*_*I*_ = *λ*_*I*_ = 21.44 GPa, *μ*_*D*_ = *λ*_*D*_ = 150.08 MPa, *θ*_0_ = 1, *Y*_0_ = 10 MPa, *Y*_1_ = 1 Pa, *a* = 60, *τ*_*I*0_ = 10^5^ s, *τ*_*D*0_ = 10^−3^ s, *β*_*I*_ = *β*_*D*_ = 0. For |*y*| > 1, the material is modified by setting *Y*_0_ = *Y*_1_ = 100 TPa to model unbreakable clamps. Thermal effects are neglected. For this test, the coefficients of the Drucker–Prager equivalent stress formula (2.7) are *A* = 1.0, *B* = 1.5 and *C* = −2.0 MPa. In figure 5 we report the computational results from an ADER-DG (*p* = 3) scheme on a uniform Cartesian mesh of 192 by 192 cells, showing good agreement with the experimental data. For a detailed mesh refinement study, see the electronic supplementary material.

### (c) Phase transition and natural convection in molten rock-like material

Seismic fault slip velocities and low thermal conductivity of rock can lead to the formation of veins of molten rock (pseudotachylytes), which are thought of as an unambiguous indicator of earthquake deformation, however, they are not common features of active faults [87]. In our model, the phase transition between solid and liquid occurs simply via the definition of the total energy by adding the contribution of the latent heat for *T* > *T*_*c*_, see (2.2), and by modifying the relaxation time for *T* > *T*_*c*_. More precisely, in this example, the relaxation time *τ*_1_ is not computed according to (2.5) and (2.6) but is considered constant (time-independent) in the solid state and is computed in terms of the dynamic viscosity *η* as $\tau_{1}=6\eta /\rho_{0}c_{s}^{2}$ for the molten state (*T* > *T*_*c*_) treated as a Newtonian fluid. Also, in this example, *θ*_1_ has to be taken as *θ*_1_ = *τ*_1_ *c*_*s*_(*ξ*)^2^/3 |***A***|^−5/3^, see the result of the asymptotic analysis presented in [63]. In the electronic supplementary material of this paper, we validate our simple approach for phase transition for a well-known benchmark problem with exact solution, namely the Stefan problem, see [88]. The obtained results clearly show that the proposed model can properly deal with heat conduction and phase transition between liquid and solid phases.

**Figure 6.**
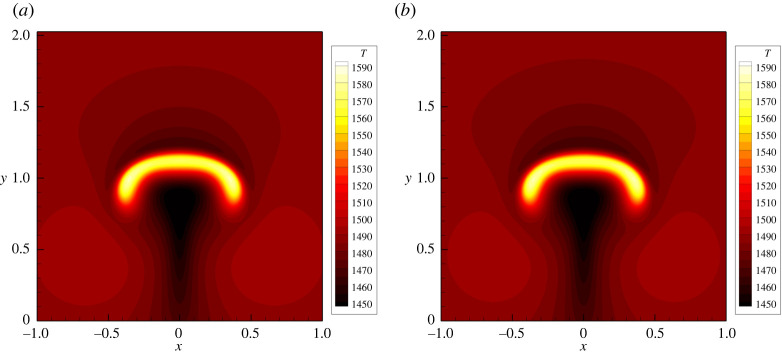
Temperature contours for the rising bubble problem in molten rock-like material at time *t* = 4. Solution obtained with the GPR model (*a*) and Navier–Stokes reference solution (*b*). The melting temperature is set to *T*_*c*_ = 1000. (Online version in colour.)

Next, we show the capability of the GPR model to describe also the motion of viscous fluids under the influence of gravity. The stresses acting on faults are key initial conditions for earthquakes and seismic fault dynamics, but are poorly known. At very long time scales, these initial conditions are governed by plate tectonics and mantle convection, which is included in the GPR model as a special case, see [89] and references therein for numerical simulations of rising bubbles in 2D and 3D. We therefore simulate a rising bubble in molten rock-like material. In the following, we use SI units. The critical temperature is set to *T*_*c*_ = 1000, the latent heat is *h*_*c*_ = 1000, the gravity vector is **g** = (0, − 9.81) and the dynamic viscosity of the molten material is *η* = 20. We furthermore set the remaining parameters to *ρ*_0_ = 2000, *γ* = 2, *p*_0_ = 2 × 10^5^, *c*_*v*_ = 0.1, *c*_*s*_ = 5, *α* = 5 and *λ* = 0.2. Initially we set *T* = 1500, *v*_*i*_ = 0, ***A*** = ***I***, ***J*** = 0, $p=10^{5}-||\text{g}||\rho_{0}y$ and a hot circular bubble of radius *R* = 0.2 is initially centred at **x**_*c*_ = (0, 0) with a temperature increase of Δ*T* = 200 for $||\text{x}-{\text{x}}_{c}||\leq R$. The domain is Ω = [ − 2, 2] × [ − 1, 3] and simulations are carried out until *t* = 4 with an ADER-DG (*p* = 3) scheme on a mesh of 200 × 200 elements. For comparison, we run two simulations, one with the GPR model presented in this paper and another simulation with the compressible Navier–Stokes equations, which serves as a reference solution for the GPR model in the viscous fluid limit. The computational results are depicted in figure 6, where we can observe an excellent agreement. This demonstrates that the model presented in this paper is also able to correctly describe natural convection in molten material when *T* > *T*_*c*_.

## 4. Summary and outlook

We have presented a unified hyperbolic model of inelasticity that incorporates finite strain elastoviscoplasticity and viscous fluids in a single PDE system, coupled with a hyperbolic model for continuous modelling of damage, including brittle and ductile fracture as particular cases. The governing equations are formulated in the Eulerian frame and via a diffuse interface approach permit arbitrary geometries of fractures and material boundaries without the necessity of generating interface-aligned meshes. We emphasize that the presented *diffuse interface* approach is not merely a way to regularize otherwise singular problems as posed by earthquake shear crack nucleation and propagation along zero-thickness interfaces, but potentially allows us to fully model volumetric fault zone shearing during earthquake rupture, which includes spontaneous partition of fault slip into intensely localized shear deformation within weaker (possibly cohesionless/ultracataclastic) fault-core gouge and more distributed damage within fault rocks and foliated gouges. The model capabilities were demonstrated in several 2D examples related to rupture processes in earthquake fault zones. We compare kinematic, fully dynamic and off-fault damage GPR diffuse rupture to models employing the traditional elasto-dynamic viewpoint of a fault, namely a planar surface across which slip occurs. We show that the continuum model can reproduce and extend classical solutions, while introducing dynamic differences (i) on the scale of pre-damaged/low-rigidity fault zone, such as out-of-plane rupture rotation, limiting peak slip rates, non-frictional control of rupture speed; and (ii) on the scale of the intact host rock, such as conjugate shear cracking in tensile lobes and amplification of velocity pulses in the emitted wavefield. A natural next step is to combine the successful micro fracture laboratory-scale Brazilian tests with dynamic rupture to span the entire scales of fault zone fracture. The GPR parameters for the host rock and fault zone rock material can also be calibrated to resemble natural rock, as e.g. Westerly granite [90]. Also, using the computational capabilities of the model’s *ExaHyPE* implementation, one can study related effects on ground shaking (see [52,66] for GPR modelling of 3D seismic wave propagation with complex topography) and detailed 3D fault zone models (cf. [91–93]) including trapped/head waves interacting with dynamic rupture [80]. Inelastic bulk processes are important during earthquake rupture (e.g. [94]), but also in between seismic events, including off-fault damage and its healing, dynamic shear localization and interseismic delocalization, and visco-elasto-plastic relaxation. Since the unified mathematical formulation proposed in this paper is able to describe elasto-plastic solids as well as viscous fluids, future work will also concern the study of fully coupled models of dynamic rupture processes triggered by mantle convection and plate tectonics. Extensions to non-Newtonian fluids will be considered, as well as to elasto-plastic saturated porous media, see e.g. the recent work presented in [65,95]. We also plan more detailed investigations concerning the onset of melting processes in shear cracks. Finally, we note that the material failure is due to the accumulation of microscopic defects (micro-cracks in rocks or dislocations in crystalline solids). It is thus interesting to remark that the distortion field being the field of non-holonomic basis triads provides a natural basis for further development of the model towards a micro-defects-based damage theory. This can be achieved via concepts of the Riemann-Cartan geometry, such as torsion discussed in [39].

## Footnotes

1 Faults are then idealized as two matching surfaces in unilateral contact not allowed to open or interpenetrate and typically implemented by splitting the fault interface [10].

2 Non-local terms can be introduced in our theory if it is physically motivated, e.g. [38,39].

3 For example, relaxation times may change over several orders of magnitude across the diffuse interface zone.

4 Global deformation can not be restored from the local triad since they represent only local deformation and thus, incompatible deformation.

## Data accessibility

*ExaHyPE* is free software hosted at www.exahype.org. The presented numerical examples will be accessible and reproducible at https://gitlab.lrz.de/exahype/ExaHyPE-Engine and https://github.com/TEAR-ERC/GPR2DR.
